# Expression Analysis of Protein Inhibitor of Activated STAT in Inflammatory Demyelinating Polyradiculoneuropathy

**DOI:** 10.3389/fimmu.2021.659038

**Published:** 2021-05-12

**Authors:** Soudeh Ghafouri-Fard, Bashdar Mahmud Hussen, Fwad Nicknafs, Naghme Nazer, Arezou Sayad, Mohammad Taheri

**Affiliations:** ^1^ Department of Medical Genetics, Shahid Beheshti University of Medical Sciences, Tehran, Iran; ^2^ Pharmacognosy Department, College of Pharmacy, Hawler Medical University, Erbil, Iraq; ^3^ Department of Electrical Engineering, Sharif University of Technology, Tehran, Iran; ^4^ Skull Base Research Center, Loghman Hakim Hospital, Shahid Beheshti University of Medical Sciences, Tehran, Iran

**Keywords:** protein inhibitor of activated STAT, PIAS, inflammatory demyelinating polyradiculoneuropathy, AIDP, CIDP

## Abstract

Protein inhibitors of activated STAT (PIAS) are involved in the regulation of the JAK/STAT signaling pathway and have interactions with NF-κB, p73 and p53. These proteins regulate immune responses; therefore dysregulation in their expression leads to several immune-mediated disorders. In the present study, we examined expression of *PIAS1*-*4* in peripheral blood of patients with acute/chronic inflammatory demyelinating polyradiculoneuropathy (AIDP/CIDP) compared with healthy subjects. We demonstrated down-regulation of all *PIAS* genes in both AIDP and CIDP cases compared with controls. Similarly, comparisons in gender-based groups revealed down-regulation of these gene0s in patients of each gender compared with gender-matched controls. There was no significant difference in expression of *PIAS* genes between AIDP and CIDP cases. Based on the area under the receiver operating characteristic curves, *PIAS1*-*4* genes could distinguish between inflammatory demyelinating polyradiculoneuropathy and healthy status with accuracy values of 0.87, 0.87, 0.79 and 0.80, respectively. In differentiation between AIDP cases and healthy controls, these values were 0.92, 0.92, 0.83 and 0.86, respectively. Finally, *PIAS1*-*4* genes could discriminate CIDP from healthy status with accuracy values of 0.82, 0.83, 0.75 and 0.75, respectively. The current study underscores the role of *PIAS* genes in the pathogenesis of inflammatory demyelinating polyradiculoneuropathy and their potential usage as biomarkers.

## Introduction

Being firstly recognized as the inhibitors of signal transducer and activator of transcription (STAT) proteins, the protein inhibitors of activated STAT (PIAS) are involved in the regulation of the JAK/STAT signaling pathway (1, 2). PIAS1, PIAS2 (PIASxα, PIASxβ), PIAS3 and PIAS4 (PIASy) are the main members of this protein family (3). In addition to the STAT family of transcription factors (TFs), PIAS proteins have functional interactions with NF-κB, p73 and p53 (4). PIAS proteins have crucial roles in the regulation of the immune responses particularly through modulation of a number of cytokine-associated genes (4). Among PIAS proteins, PIAS1 has a prominent role in the regulation of innate immune responses as it specifically suppresses expression of IFN-inducible genes. These speculations are based on the observed enhancement of the antiviral function of IFN-β and IFN-γ in Pias1−/− cells (5). PIAS2 has been shown to suppress the IL-12-associated STAT4-dependent gene induction (6). Similarly, PIAS3 has a specific inhibitory effect on STAT3 signaling (7). STAT3 has been primarily identified as a TF induced by the IL-6 family of cytokines (8). Subsequent studies revealed its essential roles in the development of Th17 cells (9), regulation of humoral immune responses (10) and modulation of innate immunity (11). PIAS4 has prominent roles in suppression of STAT1, and inhibition of LEF1 and SMAD3 signaling pathways (12). Consistent with the critical functions of PIAS proteins in the regulation of immune responses, they have been shown to be involved in the pathogenesis of multiple sclerosis (MS) (13). However, their expression pattern and functional relevance with inflammatory demyelinating polyradiculoneuropathies are largely unknown. In the current study, we examined expression of PIAS1-4 in patients with acute/chronic inflammatory demyelinating polyradiculoneuropathy (AIDP/CIDP) compared with healthy subjects. Autoimmune inflammatory responses are considered as the principal mechanisms for development of these neurologic conditions (14, 15). In addition to the pathophysiological events, these two conditions share several features in terms of clinical manifestations and treatment modalities (14). Therefore, identification of the role of *PIAS* genes in the pathogenesis of AIDP/CIDP might facilitate the process of design of therapeutic options and prediction of disease course in these conditions.

## Materials and Methods

### Enrollment of AIDP/CIDP Patients and Healthy Subjects

In total, 22 AIDP cases, 31 CIDP cases and 50 healthy individuals with no sign of inflammatory conditions were enrolled in the current study. Diagnostic evaluations were performed using the criteria offered by the American Academy of Neurology (16) and National Institute of Neurological Disorders and Stroke (17). AIDP/CIDP cases were in remission at the time of sampling. Diagnosis was based on clinical manifestations as well as electrophysiological and biochemical tests. Those with recent or chronic infection, cancer, or any systemic diseases were exempted from participation in the study. Persons enlisted in the control group were healthy individuals with no history of recent or chronic infection, malignancy, or systemic diseases. The study protocol was approved by the ethical committee of Shahid Beheshti University of Medical Sciences (IR.SBMU.MSP.REC.1398.853). All AIDP/CIDP cases and controls signed the informed consent forms.

### Expression Assay

Peripheral blood samples were obtained from controls and AIDP/CIDP patients at the time of remission. Samples were collected in EDTA-containing tubes. RNA was isolated from blood samples using the RNA extraction kit delivered by the GeneAll Company (Seoul, Korea). Then, RNA was converted to cDNA by using the Thermo Fisher Scientific kit (Brussels, Belgium). Expression levels of *PIAS* genes were measured in AIDP/CIDP cases and control subjects using the master mix supplied by the Ampliqon Company (Odense, Denmark). Expression of *PIAS* genes were measured using the Step One Plus™ Real-Time PCR system (Applied Biosystems, Foster city, CA, USA). Details of expression analysis of *PIAS* genes were explained formerly (13, 18). **Table 1** shows the primers and probes.

**Table 1:** The information about primers and probes.

Gene name	Primer and probe sequence	Primer and probe length	Product length
*HPRT1*	F: AGCCTAAGATGAGAGTTC	18	88
	R: CACAGAACTAGAACATTGATA	21	
	FAM -CATCTGGAGTCCTATTGACATCGC- TAMRA	24	
*PIAS1*	F: AGCCTAAGGGAAAGCCATAGC	21	83
	R: ATGTCTGGTATGATGCCAAAGATG	24	
	FAM-TGCCGTGTCCGTGCTGCTCCTGT- TAMRA	24	
*PIAS2*	F: CACGAACTCTTGAAGGACTTTCTG	24	126
	R: AAGTGGAAGGCAACGAGTGG	20	
	FAM - CCAGCCACGGCCAAGTCAGGTTCT -TAMRA	24	
*PIAS3*	F:GCCCTACCTGGAAGCAAAGG	20	120
	R: GTACTCATGTAGTGGGAGACTGG	23	
	FAM- CCCACCCAACGTGCCCATAGCAGG - TAMRA	24	
*PIAS4*	F: GAGAAGAAGCCCACCTGGATG	21	77
	R: AGGAGCCCGTCGATGATGAG	20	
	FAM- CCCGTGTGCGACAAGCCAGCCC - TAMRA	22	

### Statistical Methods

R programming language was used for statistical analysis. Transcript quantities of *PIAS1*, *PIAS2*, *PIAS3*, and *PIAS4* in relation to the *HPRT* reference gene was calculated from CT values using the equation:$\frac{amp_{gene}^{-CT_{gene}}}{amp_{HPRT}^{-CT_{HPRT}}}$. Then, the values were log2 transformed and used for subsequent analysis. Four comparisons between CIDP/AIDP/All patients and healthy individuals and between CIDP and AIDP patients were done and the significant difference between means was computed using the t-test. Correlations between expressions were evaluated through the calculation of Spearman correlation coefficients. Three predictive machine learning methods namely Bayesian Generalized Linear Model, Generalized Linear Model, and Linear Discriminant Analysis with 10-fold cross validation were used to compute the sensitivity and specificity of each model. The receiver operating characteristic curve was plotted. The Linear Discriminant Analysis Model (LDA) provided the most efficient estimates and in the best setting, the AUC was 0.94. Youden’s J statistic was employed to find the optimum threshold. LDA was then selected based on previous results to investigate efficiency of each gene for separating groups.

## Results

### General Demographic and Clinical Information of Cases and Controls

The current project enrolled 17 female and 36 male patients along with 13 female and 37 male subjects who enlisted as controls. Cases and controls were matched in the term of age parameter. Demographic information of patients and controls are demonstrated in **Table 2**.

**Table 2:** General demographic and clinical information of cases and controls.

Variables	AIDP Cases	CIDP cases	Controls	P value
Female/Male [no. (%)]	6 (27%)/16 (73%)	11 (35%)/20 (65%)	13 (26%)/37 (74%)	0.39
Age (mean ± SD, Y)	49.72 ± 14.6	50.5± 15.8	44.48 ± 2.3	0.13

### Expression Assays

**Figure 1:**
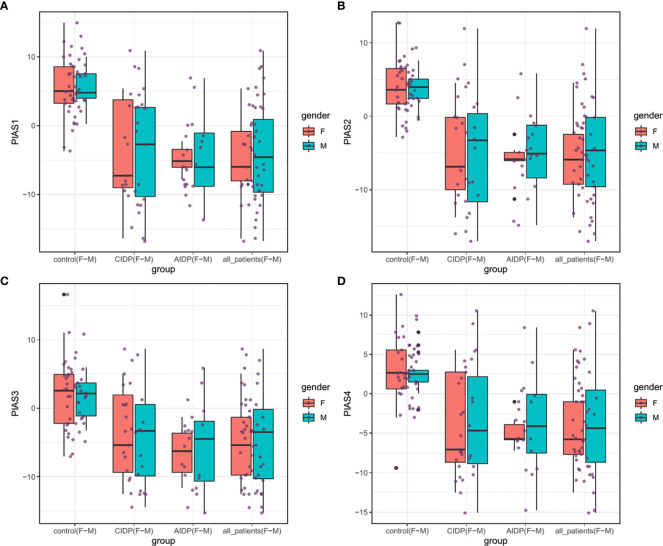
Relative expressions of *PIAS* genes in AIDP/CIDP patients and healthy subjects. Depicted box plots and whiskers show all data points from maximum to minimum. Boxes are depicted from Q1 to Q3. The horizontal lines in the middle of boxes show the median values. Each black dot shows expression in a certain sample. Mean values and interquartile range are displayed.

**Figure 1** shows the relative expression amounts of *PIAS* genes in AIDP/CIDP patients and healthy subjects. Expression levels of all *PIAS* genes were significantly decreased in both CIDP cases compared with controls. For *PIAS1*, ratio of mean expressions (RME) in cases versus controls was 1.12E-03 (P value=8.2E-14). RME (P values) for *PIAS2*, *PIAS3* and *PIAS4* genes in CIDP cases versus controls were 2.22E-03 (4.5E-13), 9.02E-03 (1.7E-08) and 1.05E-02 (3.8E-09), respectively. Expression of all genes was also decreased in AIDP cases compared with controls. The corresponding RME and P values for *PIAS1*-*PIAS4* genes in the AIDP cases versus controls were 1.52E-03 (3.3E-07), 2.60E-03 (5.9E-07), 1.47E-02 (5.9E-05) and 1.21E-02 (2.6E-05), respectively.

Similarly, comparisons in gender-based groups revealed down-regulation of these genes in patients of each gender compared with gender-matched controls. There was no significant difference in expression of *PIAS* genes between AIDP and CIDP cases. When assessing expression of *PIAS* genes in total CIDP and AIDP cases versus total controls, all genes were found to be down-regulated in affected individuals which is consistent with their expression pattern in related patients’ subgroups. **Table 3** shows the detailed statistics of expression analysis of *PIAS* genes among study groups.

**Table 3:** The results of Bayesian Regression model for comparison of expression of *PIAS* genes in AIDP/CIDP patients and healthy persons (Expressions of *PIAS* genes have been compared between CIDP cases and controls, AIDP cases and controls, CIDP cases and AIDP cases as well as total patients and total control.

Number of Samples		*PIAS1*					*PIAS2*					*PIAS3*					*PIAS4*				
		SE*	Ratio of Mean Expressions	P Value	95% CI**		SE	Ratio of Mean Expressions	P Value	95% CI		SE	Ratio of Mean Expressions	P Value	95% CI		SE	Ratio of Mean Expressions	P Value	95% CI	
CIDP/Control																					
Total	31/50	1.10	1.12E-03	**8.2E-14**	-11.99	-7.62	1.02	2.22E-03	**4.5E-13**	-10.84	-6.78	1.10	9.02E-03	**1.7E-08**	-8.98	-4.61	1.01	1.05E-02	**3.8E-09**	-8.58	-4.58
F	11/13	1.65	9.83E-04	**3.2E-06**	-13.41	-6.57	1.43	1.55E-03	**1.0E-06**	-12.28	-6.38	1.64	7.54E-03	**1.8E-04**	-10.40	-3.70	1.47	8.07E-03	**7.2E-05**	-9.98	-3.92
M	20/37	1.43	1.21E-03	**1.9E-08**	-12.58	-6.81	1.35	3.10E-03	**1.6E-07**	-11.06	-5.61	1.39	1.10E-02	**2.7E-05**	-9.30	-3.70	1.31	1.28E-02	**1.6E-05**	-8.91	-3.66
AIDP/Control																					
Total	22/50	1.53	1.52E-03	**3.3E-07**	-12.46	-6.27	1.43	2.60E-03	**5.9E-07**	-11.49	-5.68	1.38	1.47E-02	**5.9E-05**	-8.87	-3.31	1.35	1.21E-02	**2.6E-05**	-9.08	-3.65
F	6/13	2.41	1.22E-03	**1.7E-03**	-14.92	-4.44	2.02	1.98E-03	**7.7E-04**	-13.37	-4.59	2.15	1.29E-02	**1.1E-02**	-10.89	-1.66	2.09	1.06E-02	**8.1E-03**	-11.10	-2.03
M	16/37	2.00	1.73E-03	**1.1E-04**	-13.30	-5.05	1.95	3.54E-03	**3.5E-04**	-12.18	-4.11	1.77	1.76E-02	**2.8E-03**	-9.46	-2.19	1.76	1.42E-02	**1.8E-03**	-9.76	-2.52
CIDP/AIDP																					
Total	31/22	1.80	2.07E+00	5.6E-01	-2.56	4.66	1.71	1.46E+00	7.5E-01	-2.89	3.99	1.74	3.23E+00	3.4E-01	-1.80	5.18	1.64	1.43E+00	7.6E-01	-2.79	3.81
F	11/6	2.45	1.85E+00	7.2E-01	-4.42	6.20	2.26	2.00E+00	6.6E-01	-3.81	5.81	2.57	4.57E+00	4.1E-01	-3.30	7.68	2.17	2.15E+00	6.2E-01	-3.57	5.78
M	20/16	2.38	2.24E+00	6.3E-01	-3.66	5.99	2.31	1.35E+00	8.5E-01	-4.27	5.13	2.26	2.87E+00	5.0E-01	-3.07	6.11	2.18	1.25E+00	8.8E-01	-4.11	4.75
All Patients/Controls																					
Total	53/50	1.23	7.33E-04	**1.0E-09**	-12.92	-7.91	1.17	1.78E-03	**8.9E-09**	-11.51	-6.75	1.41	4.54E-03	**3.8E-06**	-10.65	-4.91	1.22	8.49E-03	**3.0E-06**	-9.36	-4.40
F	17/13	1.11	6.60E-04	**1.9E-07**	-12.95	-8.18	1.34	9.90E-04	**7.0E-05**	-13.06	-6.90	1.86	2.82E-03	**1.8E-03**	-12.75	-4.19	1.15	4.92E-03	**2.4E-05**	-10.18	-5.15
M	36/37	1.68	7.72E-04	**3.3E-06**	-13.82	-6.86	1.55	2.63E-03	**1.7E-05**	-11.79	-5.35	1.82	6.13E-03	**5.4E-04**	-11.12	-3.58	1.64	1.13E-02	**7.1E-04**	-9.86	-3.06

*SE, standard error; **95% CI, 95% confidence interval.

**Figure 2:**
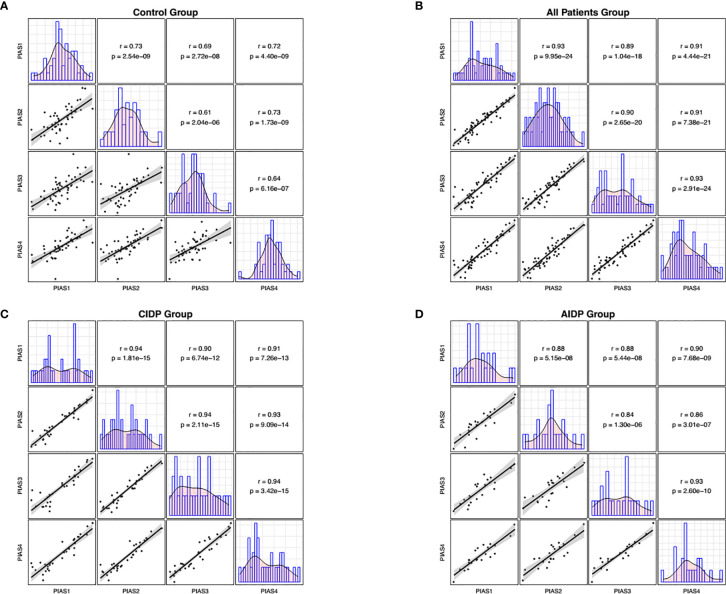
Correlations between transcript quantities of *PIAS* genes. The distributions of parameters are represented on the diagonals. The bivariate scatter plots with a fitted line are shown on the lower parts of the diagonals. Correlation coefficients and p values of the correlations are displayed on the upper sections of the diagonal.

Expression of each of PIAS genes was significantly correlated with other PIAS genes in all study groups. Notably, based on the measured correlation coefficients, such correlations were more robust among patients (**Figure 2**).

Significant P values are shown in bold letters).

**Figure 3:**
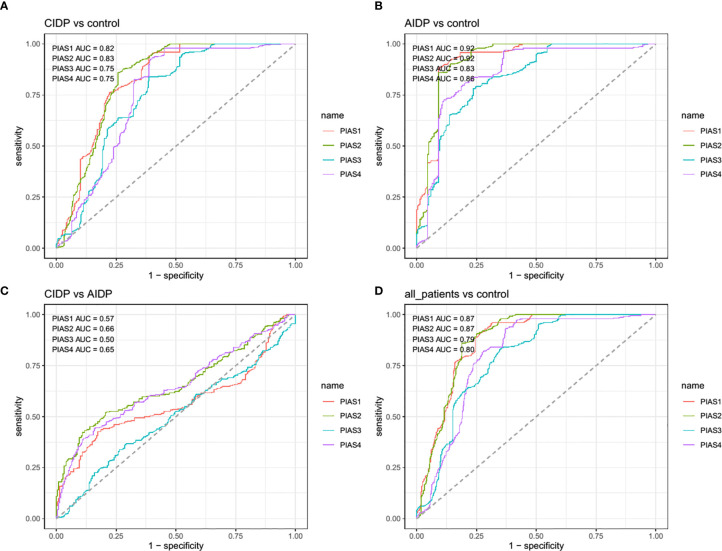
The receiver operating characteristic (ROC) curve of *PIAS1-4* in diagnosis of inflammatory demyelinating polyradiculoneuropathy.

Afterwards, we appraised whether expression of *PIAS* transcripts can separate AIDP/CIDP patients from controls (**Figure 2**). Based on the AUC values, *PIAS1*-*4* genes could distinguish between inflammatory demyelinating polyradiculoneuropathy and healthy status with accuracy values of 0.87, 0.87, 0.79 and 0.80, respectively. In differentiation between AIDP cases and healthy controls, these values were 0.92, 0.92, 0.83 and 0.86, respectively. Finally, *PIAS1*-*4* genes could discriminate CIDP from healthy status with accuracy values of 0.82, 0.83, 0.75 and 0.75, respectively. **Figure 3** and **Table 4** show details of ROC curve analysis.

**Table 4:** Detailed statistics of ROC curve analysis.

Number of Samples		*PIAS1*			*PIAS2*			*PIAS3*			*PIAS4*			All Four genes		
		AUC	Sensitivity	Specificity	AUC	Sensitivity	Specificity	AUC	Sensitivity	Specificity	AUC	Sensitivity	Specificity	AUC	Sensitivity	Specificity
CIDP/Control																
Total	31/50	0.82	0.94	0.61	0.83	0.86	0.74	0.75	0.84	0.61	0.75	0.93	0.61	0.85	0.80	0.75
AIDP/Control																
Total	22/50	0.92	0.88	0.91	0.92	0.86	0.91	0.83	0.78	0.76	0.86	0.73	0.89	0.94	0.96	0.82
CIDP/AIDP																
Total	31/22	0.57	0.43	0.83	0.66	0.52	0.80	0.50	0.37	0.71	0.65	0.44	0.84	0.49	0.68	0.47
All Patients/Controls																
Total	53/50	0.87	0.91	0.73	0.87	0.86	0.81	0.79	0.84	0.65	0.80	0.93	0.63	0.89	0.98	0.65

## Discussion

PIAS proteins have been shown to modulate the function of many TFs such as STATs, NF-κB and SMADs. The Jak-STAT cascade as a main target of PIAS proteins is the main intracellular cascade induced by class I cytokine receptor proteins. Through this signaling pathway, the activated kinase phosphorylates tyrosine amino acids in the intracellular domain of cytokine receptors (19). STATs have important roles in STATs in the development of neutrophils and regulation of their function, polarization of macrophages, and activity of dendritic cells (19). NF-κB+ signaling has also been shown to regulate expression of cytokines and antimicrobial molecules. Moreover, this signaling pathway controls differentiation, subsistence and proliferation of cells that are involved in innate and adaptive immune reactions (20). The SMAD pathway controls IgA secretion by B cells and enhances differentiation of CD4+ T cells into T17 cells and regulatory T cells (21). Therefore, PIAS proteins can affect immune responses *via* various routes. These proteins exert their regulatory roles *via* different routes such as inhibition of the DNA-binding activity of TFs and functional interaction with transcriptional corepressors or co-activators. They also have prominent roles in the regulation of innate immune reactions (4). In the current project, we demonstrated down-regulation of *PIAS1*-*4* transcripts in the peripheral blood of AIDP and CIDP patients, with no significant difference between these two groups of patients. We have recently examined expression levels of *STAT* genes in the same cohort of CIDP and AIDP patients and reported over-expression of *STAT1* in female patients compared with sex-matched controls (22). It is worth mentioning that since *PIAS1* was similarly down-regulated in males and females in the current study, this system may not be the only one responsible for elevated *STAT1* levels. STAT1 has a crucial role in induction of gene expression in response to IFN stimulation. PIAS1 is the only member of the PIAS family of proteins that can block the DNA binding function of STAT1 and suppress STAT1-associated stimulation of gene expression in response to IFN (2). Consistent with the anti-inflammatory role of PIAS1, down-regulation of this transcript has been associated with allograft rejection (23). PIAS proteins have also prominent roles in decreasing the effects of pro-inflammatory cytokines (24). Moreover, PIAS agonists and inducers have immunosuppressive effects (24). Therefore, the observed down-regulation of *PIAS* transcripts in AIDP and CIDP cases might exacerbate the autoimmune responses in these patients, thus contributing in the pathogenesis of these conditions. In line with the inhibitory role of PIAS proteins on the activity of NF-κB and the observed down-regulation of *PIAS* transcripts in AIDP/CIDP patients, Andorfer et al. have shown a remarkable increase in NF-κB levels in Guillain-Barré syndrome and CIDP cases compared to controls. They also suggested a critical role for NF-κB in the pathogenesis of these inflammatory conditions (25). Although we did not perform functional studies to assess whether down-regulation of *PIAS* transcripts is the cause or effect of CIDP/AIDP, based on the role of these transcripts in suppression of STAT signaling we hypothesize that this dysregulation is the cause of this autoimmune condition. Abnormal levels of PIAS-targeting miRNAs might be a possible mechanism of down-regulation of *PIAS* transcripts. Several miRNAs including miR-146a have been shown to affect expression of PIAS, STAT and other components of this pathway (26). Meanwhile, expression of a number of these miRNAs including miR-146a has been found to be dysregulated in GBS patients (27). Besides, miR-18a has been found to negatively regulate PIAS3 expression and therefore influencing expression of STAT3 target genes (28). Notably, miR-18a has a distinctive inhibitory effects on differentiation of T17 cells (29). Therefore, abnormal functional network between PIAS, STAT and miRNAs might affect expression and function of these pathways.

We also demonstrated significant correlations between *PIAS1-4* transcript levels particularly among patients. Such finding implies the presence of a solitary regulatory mechanism for these genes and the robustness of this mechanism in the context of AIDP/CIDP. Finally, we appraised the diagnostic power of *PIAS1*-*4* genes in distinguishing between disease and healthy conditions. The best values were detected for *PIAS1* and *PIAS2* for differentiation of AIDP cases from healthy condition.

Taken together, the observed down-regulation of *PIAS* genes in AIDP/CIDP cases implies their possible contribution in the pathogenesis of these conditions and their potential usage as biomarkers. Therapeutic options that alter expression of these genes might improve the response of patients to available therapeutic options and attenuate the course of these disorders. These speculations should be appraised in animal models of inflammatory demyelinating polyradiculoneuropathy. A limitation of this study is lack of assessment of expression levels of *PIAS* transcripts in different time points during the course of disorder to find whether their expression is changed during the time.

## Data Availability Statement

The raw data supporting the conclusions of this article will be made available by the authors, without undue reservation.

## Ethics Statement

The study protocol was approved by the ethical committee of Shahid Beheshti University of Medical Sciences (IR.SBMU.MSP.REC.1398.853). The patients/participants provided their written informed consent to participate in this study.

## Author Contributions

MT and FN performed the experiment. AS and NN collected the data and analyzed it. SG-F wrote the draft and revised it. All authors contributed to the article and approved the submitted version.

## Funding

The current study was supported by a grant from Shahid Beheshti University of Medical Sciences (Grant number 18934).

## Conflict of Interest

The authors declare that the research was conducted in the absence of any commercial or financial relationships that could be construed as a potential conflict of interest.
